# Molecular evidence for natural hybridization between wild loquat (*Eriobotrya japonica*) and its relative *E. prinoides*

**DOI:** 10.1186/s12870-014-0275-6

**Published:** 2014-10-10

**Authors:** Qiang Fan, Sufang Chen, Mingwan Li, Wei Guo, Huijuan Jing, Wei Wu, Renchao Zhou, Wenbo Liao

**Affiliations:** Guangdong Key Laboratory of Plant Resources, Key Laboratory of Biodiversity Dynamics and Conservation of Guangdong Higher Education Institutes, School of Life Sciences, Sun Yat-sen University, Guangzhou, 510275 China; Department of Horticulture and Landscape Architecture, Zhongkai University of Agriculture and Engineering, Guangzhou, 510225 China; South China Botanical Garden, Chinese Academy of Science, Guangzhou, 510650 China

**Keywords:** *Eriobotrya*, Hybridization, Nuclear genes, Chloroplast DNA

## Abstract

**Background:**

Interspecific hybridization has long been recognized as a pivotal process in plant evolution and speciation. It occurs fairly common in the genera of the subtribe Pyrinae. In *Eriobotrya*, a small tree genus of Pyrinae, *E. prinoides* var. *daduheensis* has been recognized as either a variety of *E. prinoides*, a natural hybrid between *E. prinoides* and *E. japonica*, or a variety of *E. japonica*. However, to date, there has been no convincing evidence on its status.

**Results:**

Four nuclear genes and two chloroplast regions were sequenced in 89 individuals of these three *Eriobotrya* taxa from two locations where they coexist. A few fixed nucleotide substitutions or gaps were found in each of the investigated nuclear and chloroplast loci between *E. japonica* and *E. prinoides*. Of the 35 individuals of *E. prinoides* var. *daduheensis*, 33 showed nucleotide additivity of *E. japonica* and *E. prinoides* in at least one nuclear gene, and 10 of them harboured nucleotide additivity at all the four nuclear genes. Most haplotypes of *E. prinoides* var. *daduheensis* were also shared with those of *E. japonica* and *E. prinoides*. In the two chloroplast regions, 28 and 7 individuals were identical with *E. japonica* and *E. prinoides*, respectively.

**Conclusions:**

Our study provides compelling evidence for a hybrid status for *E. prinoides* var. *daduheensis*. Most hybrid individuals are later-generation hybrids. Both *E. japonica* and *E. prinoides* can serve as female parent. Differential adaptation might maintain the species boundary of *E. prinoides* and *E. japonica* in the face of hybridization and potential introgression.

## Background

Interspecific hybridization has long been recognized as a pivotal process in plant evolution and speciation [1–3]. The understanding of the process of natural hybridization could not only help to clarify taxonomic uncertainty, but also contribute to illuminate the origin of many adaptations, the maintenance of plant diversity and the process of speciation [2]. Natural hybridization occurs fairly commonly among and within genera of the subtribe Pyrinae (formerly the Maloideae, Rosaceae), which contains many economically important fruits, such as apple, pawpaw, pear and loquat [4]. Intergeneric hybridization has been observed in 16 genera of Pyrinae [5], while intrageneric hybridization is expected to be even more frequent and has been found in many genera of Pyrinae, including *Amelanchier* [6], *Crataegus* [7–9], and *Sorbus* [10,11]. The prevalence of natural hybridization among and within the genera of Pyrinae indicates that hybridization may play important roles in the evolution of Pyrinae [4], and provides enormous opportunities to breed new cultivars of fruits.

The genus *Eriobotrya* Lindl., a small genus of Pyrinae consisting of approximately 26 species, is distributed in Himalaya, eastern Asia and western Malesia [12]. *Eriobotrya japonica* (Thunb.) Lindl., commonly known as loquat, is an important fruit tree cultivated throughout Southeastern Asia [13,14], while wild loquat is only distributed in Yunnan, Sichuan, Hubei, Guangxi and Guangdong of China [15]. Of the 21 *Eriobotrya* species found in China, there are three *Eriobotrya* species flowering in autumn except for loquat, including *E. prinoides*, *E. prinoides* var. *daduheensis* and *E. malipoensis*: *E. prinoides* Rehd. et Wils. occurs naturally in Sichuan and Yunnan, *E. prinoides* var. *daduheensis* H. Z. Zhang is distributed in two counties of Sichuan, Hanyuan and Shimian, while *E. malipoensis* occurs only in Malipo, Yunnan [15].

*E. prinoides* var. *daduheensis* has many intermediate morphological characteristics of *E. japonica* and *E. prinoides* as well as a unique pollen shape and peroxidase isozyme pattern [16]. It was later considered to be an interspecific hybrid between *E. japonica* and *E. prinoides* based on karyotype and peroxidase isozyme data [17]. In that study, all three taxa were reported as diploids with identical chromosomes (2n = 2x = 34). Additionally, the karyotype of *E. prinoides* var. *daduheensis* was either the 3A type (identical with *E. japonica*) or the 2A type (identical with *E. prinoides*), and *E. prinoides* var. *daduheensis* displayed some additivity in the peroxidase allozyme between *E. japonica* and *E. prinoides*. Further karyotype analysis by Liang et al. obtained different results in which all three taxa were the 2A type. Thus, they reconsidered *E. prinoides* var. *daduheensis* as a distinct variety of *E. japonica* [18].

These controversial results were based mostly on conventional approaches such as morphological analysis, karyotype and allozyme assay, which are far enough to provide convincing conclusions for many species showing a high degree of morphological plasticity or intermediate morphological characters arising from forces other than hybridization [19]. For most species in the subtribe Pyrinae, the frequent occurrence of polyploidization, apomixis and hybridization makes identification of hybridization events even more difficult based on these conventional approaches.

Recently, single or low-copy nuclear genes have been used successfully for identifying hybridization in plants [19–21]. The completion of the apple genome sequence provides ample ESTs for the development of exon-primed and intron-crossing (EPIC) primers to detect DNA sequence variations in the members of the subtribe Pyrinae [22–24]. In this study, more than 20 individuals of each of the three taxa (*E. prinoides* var. *daduheensis, E. japonica* and *E. prinoides*) were sampled from two locations (Hanyuan and Shimian), and four low-copy nuclear genes and two chloroplast DNA fragments were sequenced to address the following two questions: 1) Is *E. prinoides* var. *daduheensis* an interspecific hybrid? 2) If so, what is the extent of hybridization? Are these hybrid individuals F_1_s or later generation hybrids, or both? Through these efforts, we further discussed factors that may contribute to the occurrence of hybridization, the effect of hybridization on their parent species, and the possible mechanism of species integrity in the face of gene flow.

## Results

**Figure 1:**
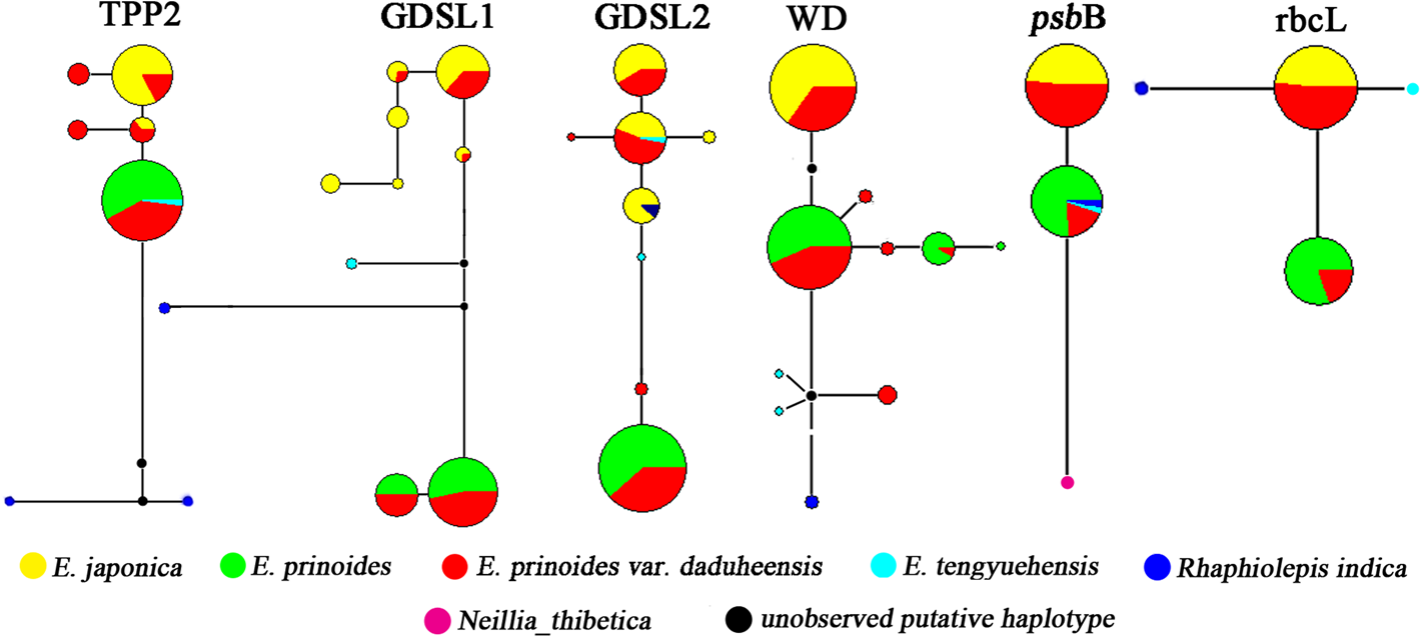
**Haplotype networks of the six investigated genes for** ***E. japonica*** **,** ***E. prinoides*** **, and their putative hybrid.** Mutational steps are shown by the length of the connecting lines, indels with more than one nucleotide were considered as one mutation, and node size is proportional to the number of haplotypes possessed by all the investigated individuals.

At these four nuclear genes and two chloroplast regions, *E. japonica* and *E. prinoides* showed fixed nucleotide substitutions. There were two fixed nucleotide substitutions at TPP2, five nucleotide substitutions and one 1-bp indel at GDSL1, four nucleotide substitutions at GDSL2, three nucleotide substitutions and one 90-bp indel at WD, and three nucleotide substitutions in the *rbc*L + *psb*B region (Table 1; Figure 1).

**Table 1:** **Fixed nucleotide substitutions and gaps between** ***E. japonica*** **and** ***E. prinoides*** **in the four nuclear genes (TPP2, GDSL1, GDSL2 and WD) and the two chloroplast genes (** ***psb*** **B and rbcL)**

**Gene**	**GDSL1**						**GDSL2**			
Site	21	229	243	292	293	595	216	238	264	562
*E. japonica*	A	A	G	G	-	A	T	C	C	A
*E. prinoides*	G	C	T	A	A	T	G	G	G	T
Gene	TPP2		WD					*psb*B		rbcL
Site	36	117	35	270–359		365	505	91	101	154
*E. japonica*	C	C	T	-		G	A	G	C	T
*E. prinoides*	T	T	G	ACA…		C	T	A	A	G

We next focused on the sites that exhibited fixed differences between *E. japonica* and *E. prinoides* for sequence analysis in *E. prinoides* var. *daduheensis*. For convenience, we designated the sequence type for each individual of *E. prinoides* var. *daduheensis* as H, J and P types if its sequence chromatogram was additive of *E. japonica* and *E. prinoides*, identical with *E. japonica*, and identical with *E. prinoides*, respectively. Of the 35 individuals of *E. prinoides* var. *daduheensis*, 33 showed nucleotide additivity of *E. japonica* and *E. prinoides* (H type) in at least at one nuclear gene (Table 2).

**Table 2:** **Sequence type of the six investigated genes in each putative hybrid** ^**a**^

**ID** ^**b**^	**Location**	**TPP2**	**GDSL1**	**GDSL2**	**WD**	***psb*** **B + rbcL**
D1	Hanyuan	H	P	H	H	J
D2	Hanyuan	H	H	H	H	J
D3	Hanyuan	H	H	H	H	P
D4	Hanyuan	J	P	H	H	J
D5	Hanyuan	J	H	H	H	J
D6	Hanyuan	H	H	H	H	J
D7	Hanyuan	H	H	H	H	J
D8	Hanyuan	J	P	H	P	J
D9	Hanyuan	P	P	H	H	P
D10	Hanyuan	P	H	J	H	P
D11	Hanyuan	H	H	H	H	P
D12	Hanyuan	H	P	H	H	P
D13	Simian	H	P	H	H	J
D14	Simian	H	P	H	P	J
D15	Simian	H	H	H	H	J
D15	Simian	H	H	H	H	J
D17	Simian	H	P	H	H	J
D18	Simian	H	P	H	H	J
D19	Simian	H	P	H	H	J
D20	Simian	H	H	H	H	J
D21	Simian	H	H	H	H	J
DP1	Hanyuan	H	P	H	P	J
DP2	Hanyuan	P	P	H	P	P
DP3	Hanyuan	H	H	H	H	P
DP4	Simian	P	P	H	H	J
DP5	Simian	H	P	P	P	J
DP6	Simian	P	P	P	P	J
DP7	Simian	H	P	P	P	J
DP8	Simian	P	J	J	P	J
DP9	Simian	P	P	H	P	J
DJ1	Simian	H	P	H	H	J
DJ2	Simian	H	J	H	J	J
DJ3	Simian	P	H	H	H	J
DJ4	Simian	H	H	P	H	J
DJ5	Simian	H	P	H	H	J

^a^According to fixed nucleotide substitutions between *E. japonica* and *E. prinoides*, sequences are same with those of *E. japonica* are defined as type “J”, sequences are same with those of *E. prinoides* are defined as type “P”, and sequences are the nucleotide additivity of both are defined as type “H”.

^b^D1-21: individual identified as typical *E. prinoides* var. *daduheensis*; DP1-9: individual with characters between *E. prinoides* var. *daduheensis* and *E. prinoides*; DJ1-5: individual with characters between *E. prinoides* var. *daduheensis* and *E. japonica*.

Among the 35 individuals of *E. prinoides* var. *daduheensis*, ten individuals were observed with H type at all four nuclear genes and 23 individuals harbored H type in at least one nuclear gene (Table 2). For the remaining two individuals, one (DP6) was P type at all four nuclear genes, and the other (DP8) was either P type or J type at the four nuclear genes. Nine of the 21 individuals with typical *E. prinoides* var. *daduheensis* characteristics (D1-D21) were observed with a H type at all four nuclear genes, while only one individual exhibited a H type at all four nuclear genes for those atypical individuals of *E. prinoides* var. *daduheensis* (DP1-DP9 and DJ1-DJ5: morphologically intermediate between *E. prinoides* var. *daduheensis* and either *E. prinoides* or *E. japonica*). At these nuclear genes, only two of these atypical individuals had a J type in two nuclear genes (Table 2).

At the two chloroplast regions, J and P types were observed in 28 and 7 individuals of *E. prinoides* var. *daduheensis*, respectively. Although DP6 was observed with P type at all four nuclear genes, it exhibited a J type at the chloroplast regions. Of the 10 individuals detected with a H type at all four nuclear genes, J and P types were observed in 7 and 3 individuals, respectively (Table 2).

For the haplotype analysis, *E. japonica* harbored a higher level of diversity than *E. prinoides* at the genes TPP2, GDSL1 and GDSL2 (2, 6 and 4 haplotypes, respectively in *E. japonica*, and 1, 2 and 1 haplotypes, respectively in *E. prinoides*). This situation is reversed at the gene WD, where *E. japonica* had only one haplotype and *E. prinoides* had three. At the chloroplast regions, no within-species variation was observed. None of these haplotypes were shared by *E. japonica* and *E. prinoides* at all four nuclear genes and two chloroplast regions. For *E. prinoides* var. *daduheensis*, most haplotypes (18/25) were the same as those of *E. japonica* or *E. prinoides* (Figure 1). There were also seven haplotypes unique to *E. prinoides* var. *daduheensis* and only one mutational step existed between the six of them and the haplotypes of *E. japonica* or *E. prinoides*.

## Discussion

### Molecular evidence for the hybrid origin of *E. prinoides* var. *daduheensis*

The taxonomic status of *E. prinoides* var. *daduheensis* has been controversial. It was recognized as either a variety of *E. prinoides* [16], a natural hybrid between *E. prinoides* and *E. japonica* [17], or a variety of *E. japonica* [18]. However, due to very limited sampling, there has been no convincing conclusion regarding its status. In this study, we aimed to characterize its status by sequencing four nuclear genes and two chloroplast regions for sufficient samples of the three taxa of *Eriobotrya* in two locations. Our results showed that there were a few fixed nucleotide substitutions between *E. japonica* and *E. prinoides* in all four nuclear genes and two chloroplast regions, indicating that the two species are well separated. Most individuals of *E. prinoides* var. *daduheensis* (33 of 35) showed nucleotide additivity of *E. japonica* and *E. prinoides* in at least one nuclear gene, providing direct evidence that they are hybrids between *E. japonica* and *E. prinoides*. The remaining two individuals, DP6 and DP8, are also hybrids because DP6 is P type at all four nuclear genes and J type at the chloroplast regions and DP8 is P type at two nuclear genes and J type at the two other nuclear genes. In the ten individuals of *E. prinoides* var. *daduheensis*, nucleotide additivity of *E. japonica* and *E. prinoides* was observed at all of the four randomly selected nuclear genes, suggesting that they might be F_1_ hybrids. Other individuals of *E. prinoides* var. *daduheensis* must be later-generation hybrids. Of the 10 putative F_1_ hybrids, J and P types were observed in 7 and 3 individuals, respectively, at the two chloroplast loci, indicating that both species could serve as female parent. In this study, seven haplotypes from 3 nuclear genes were unique to *E. prinoides* var. *daduheensis* (Figure 1). These may be due to unsampled polymorphisms from the parental species, or new mutations in the hybrids.

Molecular analyses show that 9 of 21 individuals of typical *E. prinoides* var. *daduheensis* are likely F_1_ hybrids, while there is only one out of 14 individuals of atypical *E. prinoides* var. *daduheensis*. These results suggest that typical *E. prinoides* var. *daduheensis* contains many F_1_ hybrids, whereas most atypical *E. prinoides* var. *daduheensis* are later-generation hybrids.

The four nuclear genes developed from apple, which is a Pyrinae species distantly related to *Eriobotrya*, were also successfully applied in *Eriobotrya* and *Rhaphiolepis* in this study, and other genera of Pyrinae, such as *Cotoneaster* and *Sorbus* (Chen et al. unpublished data). This indicates that the four nuclear genes can be widely applied as universal nuclear markers for hybrid identification and phylogenetic analyses in species of Pyrinae.

### Factors contributing to the natural hybridization between *E. japonica* and *E. prinoides*

The geographic distribution, flower morphology, and blooming periods of *E. japonica* and *E. prinoides* may provide ample opportunities for hybridization between these species. The two species are found in Simian and Hanyuan, Sichuan, China [15] and are partially sympatric in habitat; however, *E. japonica* prefers higher elevations and *E. prinoides* prefers lower elevations. The flowering periods of *E. japonica* and *E. prinoides* also overlap: both species flowers from October to February [16]. During our field investigations in December, 2007, we found that *E. japonica*, *E. prinoides* and *E. prinoides* var. *daduheensis* were blooming at the same time. The overall flower morphology of the two species is quite similar, except for the size of inflorescence and the number of style [16], and bees in the autumn and passerine birds in the winter are shared pollinators for them [25]. In short, *E. japonica* and *E. prinoides* have ample opportunities to hybridize with each other naturally in places where they occur together.

### Consequences of hybridization between loquat and *E. prinoides*

In this study, 25 of the 35 investigated individuals were confirmed to be later-generation hybrids, suggesting that F_1_ hybrids can backcross with parental species and introgression may take place. During our field investigations, hundreds of *E. prinoides* var. *daduheensis* individuals were found, so the potential for introgression is great. Although no signals of introgression were observed in the sampled individuals of either *E. japonica* or *E. prinoides*, the possibility cannot be excluded because only four nuclear genes and chloroplast DNA were analyzed. *E. japonica* and *E. prinoides* occupy differential altitude range with slight overlap. Differential adaptation might maintain species integrity of *E. japonica* and *E. prinoides* in the presence of hybridization and potential introgression.

Introgressive hybridization between *E. japonica* and *E. prinoides* may help to increase the genetic diversity of their wild populations, which might be advantageous for their long-term survival in the context of rapid global climate changes. Meanwhile, local people can use natural hybrid individuals to select new cultivars of loquat. Wild individuals of the two *Eriobotrya* species are widely used as rootstock to graft loquat cultivars, so many wild populations have been destroyed (Fan et al. personal observation). The current status of these *Eriobotrya* species calls for effective conservation.

## Conclusions

Based on sequence data of four low-copy nuclear genes and two chloroplast regions, we provided convincing evidence for the hybrid origin of *E. prinoides* var. *daduheensis*, and found that most hybrid individuals could be later generation hybrids. Our study demonstrated a successful application of low-copy nuclear genes in the identification of hybrids in the subtribe Pyrinae, and primers developed in this study could be applied in other genera of Pyrinae.

## Methods

### Plant sampling

In December 2007 (when the three *Eriobotrya* taxa were blooming) and in April 2008 (when the fruits of these *Eriobotrya* taxa were ripening), we conducted field surveys of the three *Eriobotrya* taxa in Hanyuan and Simian, Sichuan, China. Based on their morphological characteristics [13,16,26] and our own observations, seven diagnostic morphological characteristics were used to identify *E. japonica*, *E. prinoides* and *E. prinoides* var. *daduheensis* (Table 3). At both sampling sites, only *E. prinoides* can be found at an elevation below 800 m, whereas only *E. japonica* can be found at elevations above 1200 m. At an elevation between 800–1200 m, all three taxa can be found. In addition, some individuals exhibit an intermediate morphology of *E. prinoides* var. *daduheensis* and either *E. japonica* or *E. prinoides* (We provisionally treated them as *E. prinoides* var. *daduheensis*). In this study, we sampled 28 individuals of *E. prinoides* below 800 m, 26 individuals of *E. japonica* above 1200 m, and 35 individuals of *E. prinoides* var. *daduheensis* between 800 and 1200 m (including 21 individuals of typical *E. prinoides* var. *daduheensis*, 5 individuals with an intermediate morphology of *E. japonica* and *E. prinoides* var. *daduheensis*, and 9 individuals with an intermediate morphology of *E. prinoides* and *E. prinoides* var. *daduheensis*). In addition, one congeneric species (*E. tengyuehensis*) and one species of a closely related genus, *Rhaphiolepis indica,* were sampled and used as outgroups (Table 4). For the *psb*B gene, a sequence from *Neillia thibetica* (Rosaceae) was downloaded from NCBI website and set as outgroup (Accession number: JF317470). All of the leaves for DNA extraction were collected and stored in silica gel in zip-lock plastic bags until use. Voucher specimens were deposited in the Herbarium of Sun Yat-sen University (SYS).

**Table 3:** **Morphological characters of** ***E. japonica*** **,** ***E. prinoides*** **and typical** ***E. prinoides*** **var.** ***daduheensis***

**Character**	***E. japonica***	***E. prinoides***	***E. prinoides*** **var.** ***daduheensis***
Leaf margin	Serrate	Undulate	Serrate
Upper surface of leaf	Rugose	Smooth	Smooth
Leaf size (cm)	12–30 × 5–10	7–15 × 3.5–7.5	10–24 × 4.5–9
Petiole length (cm)	0.2–0.8	1.5–3	1–2.5
Stipule shape	Subulate	Ovate	Subulate
Inflorescence length (cm)	10–20.5	6–10	8–12
Number of styles	5	2, rarely 3	3–4

**Table 4:** **Sample locations of** ***E. japonica*** **,** ***E. prinoides*** **,** ***E. prinoides*** **var.** ***daduheensis*** **and outgroups used in this study**

***E. japoncina*** **(J),** ***E. prinoides*** **(P), and** ***E. prinoides*** **var.** ***Daduheensis*** **(H)**				
		**J**	**P**	**H**
Shimian County, Sichuan, China	29°14′N, 102°19′E	13	13	20
Hanyuan County, Sichuan, China	29°17′N, 102°39′E	13	15	15
Other species used as outgroup				
Gongshan, Yunnan, China	27°43′N, 98°40′E	*E. tengyueensis*		
Guangzhou, Guangdong, China	23°6′N, 113°18′E	*Rhaphiolepis indica*		

### DNA extraction, primer design, PCR and sequencing

Genomic DNA was isolated using the CTAB method [27]. Based on them, specific EPIC primers were designed using Primer Premier 6.0 (PREMIER Biosoft International, Palo Alto, CA, USA). These cDNAs encode tripeptidyl peptidase II (TPP2), GDSL esterase/lipase APG-like proteins (GDSL), and WD repeat-containing protein (WD). One bright band was amplified for the TPP2 and WD primers, and two bright bands were amplified using the GDSL primers (we designated them as GDSL1 and GDSL2). The chloroplast *rbc*L region was amplified using universal primers [28], while a partial *psb*B region was amplified using specific primers based on the sequences of some individuals of *Eriobotrya*, which were obtained from the universal primers *psb*B1-F and *psb*B2-R [29]. All of the primer sequences are listed in Table 5.

**Table 5:** **Primers of the four nuclear genes designed from a coding sequence database of** ***Malus*** **×** ***domestica***

**Locus**	**Primer sequences (5′-3′)**	**The aligned length (bp)**	**ID for apple coding sequence**
TPP2	F: GCTGGTTTTGTTCATCGA	389	MDP0000193152
	R: ACCGCTCAGAAACAGGCT		
GDSL1	F: GTCTTCCAAGGCTTCGTT	661	MDP0000137339
	R: ACAATTCCCGTTCCACAG		
GDSL2	F: identical with GDSL1	591	identical with GDSL1
	R: identical with GDSL1		
WD	F: GTTCCTCTATCATCACCAGTT	736	MDP0000283138
	R: ACCAGTGCCAAGTCTATTC		
rbcL	724F: GCTACTGCAGGTACATG	754	
	1352R: CTTCACAAGCAGCAGCTAGTTC		
*psb*B	F:AACGAGTGGGACCAAATA	317	
	R:TTTCTATTCAGGGGTGGC		

PCR amplifications were performed in 20 μL reaction volumes, containing 25 ng of genomic DNA, 2 μL of 10 × buffer (with Mg^2+^), 0.25 mM of dNTPs, 0.2 μM of each primer, and 1 U of Easy-Taq DNA polymerase (TransGen Biotech Co., Ltd, Beijing, China). The PCR reactions were conducted with the following conditions: initial denaturing at 94°C for 2.5 min, followed by 35 cycles of 94°C for 30 s, a corresponding annealing temperature (50°C for TPP2, WD, and *rbc*L, and 55°C for GDSL and *psb*B) for 30 s, 72°C for 1 min, and a final extension at 72°C for 5 min. The PCR products were purified by electrophoresis with a 1.2% agarose gel followed by the use of a Pearl Gel Extraction Kit (Pearl Biotech, Guangzhou, China). Following purification, they were sequenced on an ABI 3730 DNA automated sequencer with the BigDye Terminator Cycle Sequencing Ready Reaction Kit (Applied Biosystems, Foster City, CA). All sequences were deposited in GenBank under accession numbers KF699531-KF699842, KJ735102-KJ735383, and KM246943-KM246944.

### Sequences analysis

The sequences were assembled and edited with SeqMan™ II (DNASTAR, Inc., Madison, WI), and then subjected to a BLASTN search against the genome sequence of apple (http://www.rosaceae.org/species/malus/malus_x_domestica/genome_v1.0) to determine their possible copy number in the genome, setting an E-value cut-off of 1e^-6^ and a minimum score of 100 bits. The results showed that no more than three hits were obtained for each of the four investigated genes, indicating that they are low-copy ones in the genome. For each of the four nuclear genes, we also designed 2–3 pairs of primers anchoring different locations (data not shown). Sequencing for their PCR products produced identical sequences at the target regions, supporting that they are very likely orthologous in *Eriobotrya*. Polymorphisms at variable sites were identified as superimposed nucleotide additivity patterns from chromatograms of direct sequencing [30], and indels were identified by reading the sequence chromatogram from both sides. The haplotype inference of the four nuclear genes was implemented with PHASE v2.1 [31,32]. The haplotype network was constructed for each gene using Network 4.6.1.2 (www.fluxus-engineering.com) with the median-joining algorithm [33].

## References

[1] Mallet J (2007). Hybrid speciation. Nature.

[2] Soltis PS, Soltis DE (2009). The role of hybridization in plant speciation. Annu Rev Plant Biol.

[3] Hegarty MJ, Hiscock SJ (2005). Hybrid speciation in plants: new insights from molecular studies. New Phytol.

[4] Campbell CS, Evans RC, Morgan DR, Dickinson TA, Arsenault MP (2007). Phylogeny of subtribe Pyrinae (formerly the Maloideae, Rosaceae): limited resolution of a complex evolutionary history. Plant Syst Evol.

[5] Robertson KR, Phipps JB, Rohrer JR, Smith PG (1991). A synopsis of genera of the Maloideae (Rosaceae). Syst Bot.

[6] Campbell CS, Wright WA (1996). Apomixis, hybridization, and taxonomic complexity in eastern North American Amelanchier. Folia Geobot Phytotax.

[7] Byatt JI (1975). Hybridization between *Crataegus monogyna* Jacq. **and***C. laevigata* (Poiret) DC. in south-eastern England. Walsonia.

[8] Gosler AG (1990). Introgressive hybridization between *Crataegus monogyna* Jacq. and *C. laevigata* (Poiret) DC. in the Upper Thames Valley, England. Watsonia.

[9] Phipps JB (2005). A review of hybridization in north American hawthorns - another look at “the Crataegus problem”. Ann Mo Bot Grad.

[10] Robertson A, Rich TC, Allen AM, Houston L, Roberts C, Bridle JR, Harris SA, Hiscock SJ (2010). Hybridization and polyploidy as drivers of continuing evolution and speciation in *Sorbus*. Mol Ecol.

[11] Ludwig S, Robertson A, Rich TC, Djordjevic M, Cerovic R, Houston L, Harris SA, Hiscock SJ (2013). Breeding systems, hybridization and continuing evolution in Avon Gorge *Sorbus*. Ann Bot.

[12] Mabberley DJ (1997). The Plant-Book: A Portable Dictionary of the Vascular Plants.

[13] Gu CZ, Spongberg SA, Wu CY, Raven PH, Hong DY (2003). *Eriobotrya*. Flora of China. Volume 9.

[14] Qiu WL, Zhang HZ (1996). Fruit Flora of China (Longan and Loquat).

[15] Lin SQ, Yang XH, Liu CM, Hu YL, He YH, Hu GB, Zhang HL, He XL, Liu YX, Liu ZL (2004). Natural geographical distribution of genus *Eriobotrya* plants in China. Acta Horti Sin.

[16] Zhang HZ, Peng S, Cai LH, Fang DQ (1990). The germplasm resources of the genus *Eriobotrya* with special reference on the origin of *E. japonica* Lindl. Acta Horti Sin.

[17] Tang B (1997). A study of the sibship among *Eriobotrya japonica* Lindl, *E. prinoides* Rehd & wils. var. *daduheensis* H. Z. Zhang and *E. prinoides* Rehd. & Wils. Journal of Chongqing Teachers College (Natural Science Edition).

[18] Liang GL, Ren ZC, Yan Y, Li XL, Jiang NG (2001). Karyotype variation and evolution of genus *Eriobotrya* in Sichan. Adv Chromosome Sci.

[19] Zhang R, Liu T, Wu W, Li Y, Chao L, Huang L, Huang Y, Shi S, Zhou R (2013). Molecular evidence for natural hybridization in the mangrove fern genus *Acrostichum*. BMC Plant Biol.

[20] Wu W, Zhou R, Huang Y, Boufford DE, Shi S (2010). Molecular evidence for natural intergeneric hybridization between *Liquidambar* and *Altingia*. J Plant Res.

[21] Zha HG, Milne RI, Sun H (2010). Asymmetric hybridization in *Rhododendron agastum*: a hybrid taxon comprising mainly F_1_s in Yunnan, China. Ann Bot.

[22] Velasco R, Zharkikh A, Affourtit J, Dhingra A, Cestaro A, Kalyanaraman A, Fontana P, Bhatnagar SK, Troggio M, Pruss D, Salvi S, Pindo M, Baldi P, Castelletti S, Cavaiuolo M, Coppola G, Costa F, Cova V, Dal Ri A, Goremykin V, Komjanc M, Longhi S, Magnago P, Malacarne G, Malnoy M, Micheletti D, Moretto M, Perazzolli M, Si-Ammour A, Vezzulli S (2010). The genome of the domesticated apple (*Malus*x domestica Borkh.). Nat Genet.

[23] Thomson RC, Wang IJ, Johnson JR (2010). Genome-enabled development of DNA markers for ecology, evolution and conservation. Mol Ecol.

[24] Zhou R, Zeng K, Wu W, Chen X, Yang Z, Shi S, Wu CI (2007). Population genetics of speciation in nonmodel organisms: I. Ancestral polymorphism in mangroves. Mol Biol Evol.

[25] Fang Q, Chen YZ, Huang SQ (2012). Generalist passerine pollination of a winter-flowering fruit tree in central China. Ann Bot.

[26] Kuan KC, Yü TT, Yü TT (1974). *Eriobotrya*. Rosaceae (1) Flora Reipublicae Popularis Sinicae. Volume 36.

[27] Doyle JJ, Doyle JL (1987). A rapid DNA isolation procedure from small quantities of fresh leaf tissues. Phytochem Bull.

[28] Fay MF, Swensen SM, Chase MW (1997). Taxonomic affinities of *Medusagyne oppositifolia* (Medusagynaceae). Kew Bull.

[29] Heinze B (2007). A database of PCR primers for the chloroplast genomes of higher plants. Plant Meth.

[30] Whittall J, Liston A, Gisler S, Meinke RJ (2000). Detecting nucleotide additivity from direct sequences is a SNAP: an example from *Sidalcea* (Malvaceae). Plant Biol.

[31] Stephens M, Smith NJ, Donnelly P (2001). A new statistical method for haplotype reconstruction from population data. Am J Hum Genet.

[32] Stephens M, Scheet P (2005). Accounting for decay of linkage disequilibrium in haplotype inference and missing-data imputation. Am J Hum Genet.

[33] Bandelt HJ, Forster P, Rohl A (1999). Median-joining networks for inferring intraspecific phylogenies. Mol Biol Evol.

